# Relevance of the Updated Recursive Partitioning Analysis (U-RPA) Classification in the Contemporary Care of Patients with Brain Metastases

**DOI:** 10.3390/cancers15123255

**Published:** 2023-06-20

**Authors:** Camilo E. Fadul, Guneet Sarai, Joseph A. Bovi, Alissa A. Thomas, Wendy Novicoff, Roger Anderson, Ryan F. Amidon, Samantha Schuetz, Rohit Singh, Amy Chang, Ryan D. Gentzler, Elizabeth M. Gaughan, Jason P. Sheehan

**Affiliations:** 1 Division of Neuro-Oncology, Department of Neurology, University of Virginia School of Medicine, Charlottesville, VA 22908, USA; 2 Department of Radiation Oncology, The Medical College of Wisconsin, Milwaukee, WI 53222, USA; jbovi@mcw.edu (J.A.B.);; 3 Department of Neurological Sciences, University of Vermont Larner College of Medicine, Burlington, VT 05405, USA; 4 Department of Public Health Sciences and Orthopedic Surgery, University of Virginia School of Medicine, Charlottesville, VA 22908, USA; 5 Population Sciences, University of Virginia School of Medicine, Charlottesville, VA 22908, USA; 6 Division of Hematology and Oncology, University of Vermont Larner College of Medicine, Burlington, VT 05405, USA; 7 Division of Hematology and Oncology, Department of Medicine, University of Virginia School of Medicine, Charlottesville, VA 22908, USA; 8 Department of Neurological Surgery, University of Virginia School of Medicine, Charlottesville, VA 22908, USA

**Keywords:** brain metastases, whole brain radiation therapy, stereotactic radiosurgery, brain-directed treatment, recursive partitioning analysis, Karnofsky performance status, prognosis

## Abstract

**Simple Summary:**

Brain metastases are the most frequent type of intracranial cancer. Although the prognosis has improved, the outcomes still vary widely, with a median survival of less than one year. The Recursive Partitioning Analysis (RPA) is one of the first proposed brain metastases prognostic tools. We hypothesized that an Updated RPA (U-RPA) prognostic index, which can easily be determined in the clinic, would be informative for both patients and clinicians to make decisions in the contemporary management of brain metastases. We report a retrospective analysis of all patients treated at three academic institutions, between 2017 and 2019, to compare outcomes based on the U-RPA and according to the type of treatment and primary cancer. Our results suggest that determination and documentation of the U-RPA at the time of diagnosis may assist patients and clinicians to make better-informed decisions based on the potential value of contemporary brain-directed treatment options.

**Abstract:**

Patients with brain metastases (BMETS) need information about the prognosis and potential value of treatment options to make informed therapeutic decisions, but tools to predict survival in contemporary practice are scarce. We propose an Updated Recursive Partitioning Analysis (U-RPA) instrument to predict survival and benefit from brain-directed treatment (BDT) of contemporary patients. This was a retrospective analysis of patients with BMETS treated between 2017 and 2019. With survival as the primary endpoint, we calculated the U-RPA and generated estimates using Kaplan–Meier curves and hazard ratios. Of 862 eligible patients, 752 received BDT and 110 received best supportive care (BSC). Median overall survival with BDT and BSC was 9.3 and 1.3 months, respectively. Patients in RPA class 1, 2A, 2B and 3 who underwent BDT had median survival of 28.1, 14.7, 7.6 and 3.3 months, respectively. The median survival for patients in RPA 3 who received BDT (*n* = 147), WBRT (*n* = 79) and SRS (*n* = 54) was 3.3, 2.9 and 4.1 months, respectively. The U-RPA defines prognosis estimates, independent of tumor type and treatment modality, which can assist to make value-based care treatment decisions. The prognosis for patients in U-RPA class 2B and 3 remains poor, with consideration for early palliative care involvement in these cases.

## 1. Introduction 

The incidence of brain metastases (BMETS) is unknown, but epidemiologic studies suggest that there are at least 70,000 new cases diagnosed in the US every year [1,2]. Although the prognosis is generally poor with a median survival of less than a year, more effective treatment strategies have improved outcomes for selected patients [3]. Ranges of survival are broad, with some patients surviving less than 90 days and other living for years [3]. Patients with BMETS may lack prognostic awareness and clear understanding of potential outcomes to make informed treatment decisions [4,5], while clinicians may be inaccurate in their prognostic predictions, providing an overly optimistic benefit from proposed therapies [6]. Therefore, patients and clinicians need to be aware of prognostic estimates and the trade-off between the potential benefit and adverse effects (value) of the available treatment options, including palliative care, to make an informed decision. 

Several BMETS prognostic indices that have been proposed and studied have had the main objective of determining eligibility for clinical trials and of supporting treatment decisions but which one would be the most valuable in routine clinical practice is uncertain [7]. The most frequently cited instruments are the Recursive Partitioning Analysis (RPA) and the Graded Prognostic Assessment (GPA) [8,9,10]. Characteristics of the ideal prognostic index include determination from objective criteria, ease of use and accurate predictive survival value according to cancer type and treatment received in the era of immunotherapy and tyrosine kinase inhibitors. A reliable prognostic estimate at the time of diagnosis remains crucial to guide shared treatment decisions and provide value-based care, but documentation of a prognostic index is not the standard practice in the current care of patients with BMETS. 

While the GPA classification has more detailed, disease-specific information, the RPA classification has the advantage of being tissue-agnostic and using limited consistent variables (age, Karnofsky performance status (KPS), primary tumor control and extracranial metastases) [9], so it can be used to start framing conversations around treatment decisions from the moment of initial presentation. We hypothesized that an Updated RPA (U-RPA) prognostic index, which subclassified the patients in class 2 according to their KPS and can easily be determined in the clinic, would be informative for both patients and clinicians to make decisions in the modern-day management of BMETS. If the U-RPA has the potential to assist patients and clinicians on complex treatment decisions, its documentation would be relevant in routine clinical practice. We conducted a retrospective quality analysis of all patients treated at three academic institutions, between 2017 and 2019, to provide a real-world application of the U-RPA classification in a contemporary cohort of patients with BMETS.

## 2. Methods

### 2.1. Study Population

We obtained data from the electronic medical record (EMR) from all patients diagnosed with BMETS and treated at the University of Virginia (UVA), the Medical College of Wisconsin (MCW) and the University of Vermont (UVM) between 1 January 2017 and 31 December 2019. Eligible patients were age 18 years or older and had a diagnosis of solid tumor malignancy, and if they did not die before, they had at least six months of follow-up of treatment of BMETS at the participating institution. Patients with leptomeningeal or dural/calvarial metastases were excluded. 

### 2.2. Updated-RPA Prognostic Index

Collected data included patient age, sex, race, KPS at the time of BMETS diagnosis, type of primary solid tumor, targetable genomic alteration for lung adenocarcinoma and melanoma (EGFR, ALK or BRAF) and type of BMETS treatment received, including surgery, WBRT, SRS, systemic medical therapy and best supportive care (BSC). Patients who had surgery were only included in the BDT group if resection was followed by either radiation or systemic therapy within 60 days of the procedure. We defined survival as the interval between the date of BMETS diagnosis based on imaging studies and date of death, if applicable, or to the last date of follow-up if the patient was still alive or lost to follow-up. 

We calculated the RPA based on age, documented KPS and review of central nervous system (CNS) and extra-cranial disease status based on imaging results and clinical documentation at the time of brain metastases diagnosis. As described by Gaspar et al. [9], we divided patients into: class 1 (patients with KPS greater than or equal to 70, age less than 65 years, controlled primary and no extracranial metastases), class 3 (patients with KPS less than 70) and class 2 (all other patients). According to the KPS, we further subclassified the patients in class 2 into 2A (KPS 90–100) and 2B (KPS 70–80) and designated the four classes as the Updated-RPA (U-RPA). We chose to use Arabic instead of Roman numerals for this prognostic index to avoid confusion. 

### 2.3. Statistical Analysis

We performed descriptive statistics for all demographic variables. Binary logistic regression was used to examine variables related to survival status, with validation of the logistic regression model done using a randomly selected 30% sample (there was no overlap between the testing sample and the validation sample). Odds ratios with their 95% confidence intervals were computed for each variable in the regression model, and the Receiver Operating Characteristic (ROC) Curve was generated for the overall model. We generated Kaplan–Meier survival curves for all patients and for several subgroups based on U-RPA and treatment modality; the log-rank test was used to compare survival curves for each subgroup, with significance set at *p* < 0.01. We calculated the hazard ratios for the U-RPA survival curves in univariate and multivariate analysis using U-RPA class 1 as the control. We performed the statistical analysis using GraphPad Prism version 9.0.0 for Windows (GraphPad Software, San Diego, CA, USA) and SPSS Version 28 (IBM Corp. Released 2021. IBM SPSS Statistics for Windows, Version 28.0. Armonk, NY, USA).

## 3. Results 

We included 862 consecutive eligible patients with a follow-up of at least 6 months or until death. At the time of analysis with a minimum follow-up of 24 months, 17 (2%) patients were lost to follow-up, and 135 (16%) remained alive. Table 1 shows the patient characteristics and treatment received. The most frequent primary cancers were non-small cell lung cancer (NSCLC), breast, melanoma and small cell lung cancer (SCLC), which accounted for 75% of all BMETS origin. In 62 (16%) of the 393 patients with NSCLC, the tumor had EGFR (46, 12%), ALK (8, 2%) or BRAF V600E (8, 2%) targetable genomic alterations. Of the 93 patients with melanoma, 35 (38%) had a BRAF V600E mutation.

**Figure 1:**
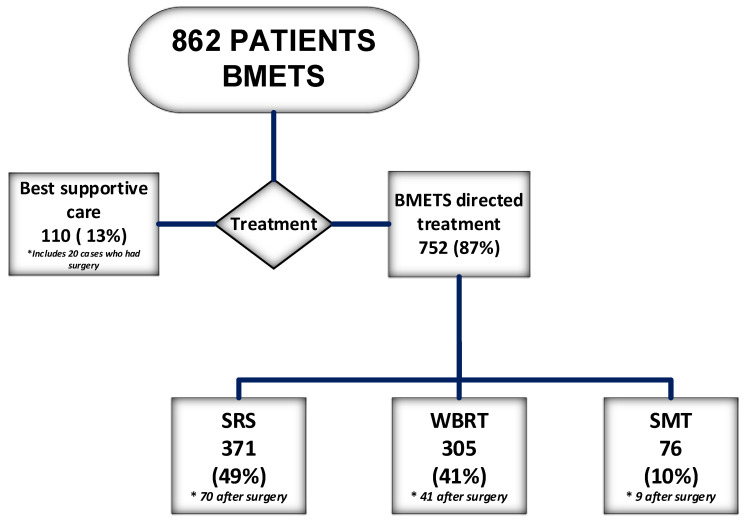
Management of 862 patients diagnosed with brain metastases 2017–2019. * Indicates the number of patients in each group who had surgery.

Of the 862 patients, 752 (87%) received brain-directed treatment (BDT) and 110 (13%) received BSC. Figure 1 shows the proportion of patients receiving each type of BDT. Of the 140 patients who had BMETS resection, 120 (included in the BDT group) had another type of treatment, which was SRS in 70 (58%), WBRT in 41 (34%) and systemic medical therapy alone in nine (8%); the other 20 patients either received BSC or died before receiving BDT. The median OS for all patients was 7.0 months, 9.3 months for 752 patients (87%) who received BDT and 1.3 months for 110 patients (13%) who received BSC. The median survival for patients who received WBRT, SRS or resection followed by radiation or systemic medical therapy was 4.9, 12.9 and 14.3 months, respectively. Approximately 26% of patients who received BDT were alive at 2 years.

**Figure 2:**
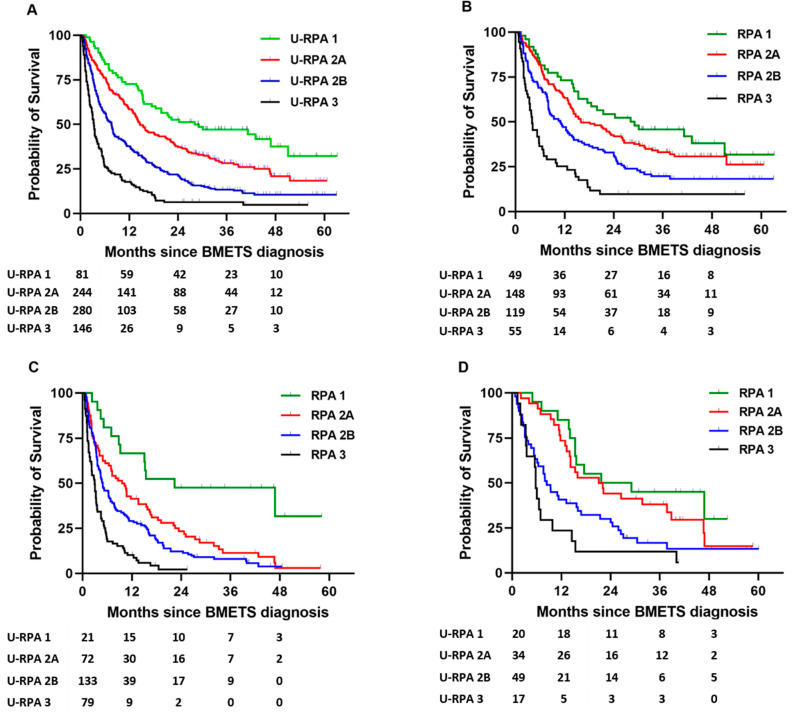
Kaplan–Meier survival curves for (**A**) all patients, (**B**) patients treated with SRS, (**C**) patients treated with WBRT and (**D**) patients treated with surgery followed by BDT.

Of the 752 patients receiving BDT, 81 (11%) were RPA 1, 524 (69%) were RPA 2 and 148 (20%) were RPA 3. We further divided the 524 patients in RPA 2 by their KPS into 2A (*n* = 244) and 2B (*n* = 280). Table 2 compares the median survivals of patients who received BDT according to RPA and U-RPA. There was significant difference in the survival curves according to U-RPA class for all patients who received BDT (log-rank *p* < 0.001) and according to treatment modality: SRS (log-rank *p* < 0.001), WBRT (log-rank *p* < 0.001) and surgery followed by BDT (log-rank *p* = 0.002) (Figure 2). Of 84 patients in RPA class 1, 81 (96%) received BDT with median survival of 28.1 months. Of 259 patients in class 2A, 244 (94%) received BDT with a median survival of 14.7 months. Of 310 patients in RPA class 2B, 280 (90%) received BDT with a median survival of 7.6 months. Of 208 patients in RPA class 3, 148 (71%) received BDT with a median survival of 3.3 months. The survival of U-RPA class 2B with WBRT (*n* = 133) was 4.9 months and for SRS (*n* = 119) was 10.9 months (Table S1). The median survival of patients in U- RPA class 3 receiving WBRT (*n* = 79) was 2.9 months and for those receiving SRS (*n* = 54) was 4 months.

**Figure 3:**
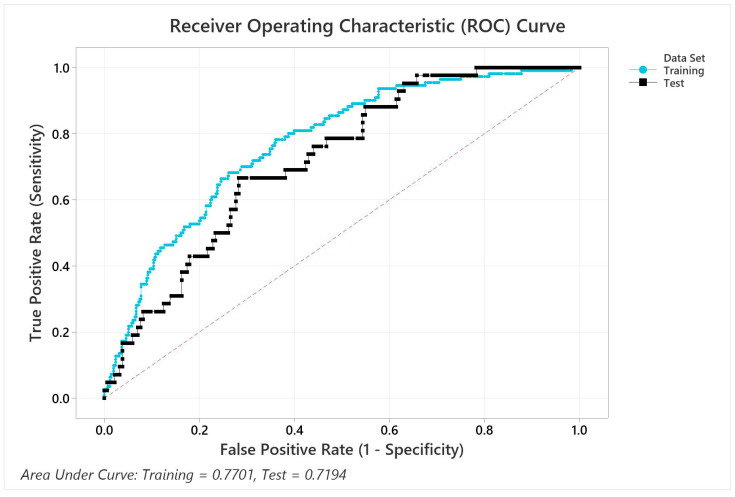
Receiver Operating Characteristic Curves for the training and validation sets.

Using a binary regression model, we found that the U-RPA was the most significant variable associated with survival (*p* = 0.004). In univariate analysis, other variables associated with survival and included in the model were age at diagnosis (*p* = 0.035), KPS score (*p* = 0.032) and treatment (*p* = 0.032). In multivariate analysis, using the same variables as in the model, the HR for U-RPA classes 2A, 2B and 3 were significantly higher than class 1 (Table 3). Figure 3 shows the ROC curves with an area under the curve for the training set of 0.7701 and the validation set of 0.7104.

The median survival for patients who received BDT for BMETS from NSCLC (*n* = 345), melanoma (*n* = 86) and breast cancer (*n* = 83) were 11.5 months, 10.4 months and 11.1 months, respectively. We created Kaplan–Meier survival curves for the most frequent primary cancers, including NSCLC patients with no genomic alterations, according to the U-RPA (Figure S1). Table 4 shows the median survival times by primary; all had log-rank *p*-values of <0.001. The median survival for patients with RPA class 3 and BMETS from NSCLC, melanoma and breast cancer were 3.5 months, 2.3 months and 3.2 months, respectively.

We compared the survival curves for all patients as well as for those who received SRS or WBRT according to treating institution (Figure S2). The median survival times for patients treated at UVM, UVA and MCW were 6.3, 8.0 and 6.3 months, respectively (log-rank *p* = 0.2071). The median survival times for patients treated with SRS or WBRT at UVM, UVA and MCW were 7.0, 9.7 and 9.3 months, respectively (log-rank *p* = 0.3353).

## 4. Discussion

Although survival of patients with BMETS has improved, their outcomes still vary widely, underlining the need for an index that allows predictions of outcomes in the setting of therapeutic advances. We found that the RPA, one of the first proposed prognostic tools, remains a valuable instrument to provide survival estimate on contemporary patients with BMETS independent of the treatment modality and primary cancer. As most patients corresponded to RPA class 2, we sub-classified them according to KPS into 2A and 2B for a prognostic index with four distinct survival classes, the U-RPA. We show that the prognosis for modern-day patients receiving BDT has strikingly improved, especially in U-RPA class 1 (median OS 28.1 months) and 2A (median OS 14.7 months) compared to the initial RPA publication, but for those in class 3 the median survival remains at about 3 months. Our findings suggest that use of the U-RPA in current clinical practice may allow patients and clinicians to make better-informed decisions on the potential value of brain-directed treatment options.

On our review of the records of 852 contemporary patients treated in three academic institutions, there was no consistent documentation of a BMETS-specific prognostic tool assessment in the EMR. Estimation of the prognosis for patients with BMETS influences treatment decisions, but clinicians are inaccurate on appraising their survival [6,11]. Patients have the perception that there is need for more information about the prognostic and therapeutic implications at the time of diagnosis [4,5], and both patients and clinicians may be overly optimistic about the outcome [5,6,11]. Our study suggests that to make a shared informed decision about treatment, the U-RPA may be a convenient and reliable tool in the modern era to convey prognostic estimates at the time of BMETS diagnosis.

We found that by dividing patients in RPA class 2 according to their KPS, two distinct survival curves allowed better prognostic accuracy with median survival of 14.7 months for 2A (KPS 90–100) and 7.6 months for 2B (KPS 70–80). A previous study proposed to divide patients in class 2 treated with SRS into three subclasses according to a score obtained from four factors: KPS, number of BMETS, control of primary diseases and presence of extra-neural metastases [12]. The U-RPA relies mainly on the functional status, which allows easy and consistent determination by the clinician, while providing patients with a general understanding about their prognosis. A comparison of prognostic scales in a cohort of patients treated with SRS in the modern era revealed that the RPA and an index based on performance status performed similar to other scales in predicting survival [13]. Although scales that provide primary cancer-specific prognostic groups, such as the updated disease-specific GPA tool [8], may be more accurate, we showed that the U-RPA provides survival estimates according to class that are similar to the most frequent neoplasms. Patients with NSCLC, melanoma and breast cancer primaries in U-RPA class 1 had a median survival between 43 and 47 months, while for those on class 3, the survival was between 2.3 and 3.5 months. Potential advantage of the U-RPA is that it provides an easily determined estimate on survival according to BDT modality to inform patients and clinicians at the time of diagnosis about the potential value from treatment.

Our results reflect the significant improvement in survival of patients with BMETS receiving contemporary treatment except for the 71% of those in RPA class 3 who received BDT. Their median survival of 3.3 months is similar to that reported in the original RPA publication of 2.3 months [9]. A reason to recommend BDT for these patients may be improvement in neurologic symptoms and quality of life. There has been no evidence of improvement in survival or quality of life of WBRT or SRS in patients with BMETS and U-RPA 3 [14,15]. However, 61% of our real-world contemporary patients in U-RPA class 3 treated in academic centers received WBRT or SRS. The proportion of patients in class 3 for whom BSC would be of more value than BDT may be higher than the 30% in our series. Therapeutic decisions for patients in U-RPA class 3 may require a personalized multidisciplinary assessment that includes palliative care consultation to provide value-based care and weigh the potential harms from overtreatment or futility.

Whole brain radiation was the treatment for 41% of patients who received BDT resulting in a median survival of 4.9 months. Excluding patients in U-RPA class 3, the median survival after WBRT in our cohort was 6.8 months, which is similar to the one reported for both arms in the phase III trial comparing hippocampal avoidance-WBRT (6.3 months) or WBRT (7.6 months) plus memantine [16]. The number of patients receiving WBRT for BMETS in the US is unknown, but a recent survey showed that non-academic physicians were more likely to recommend WBRT for patients with BMETS [5]. The QUARTZ study revealed that WBRT for patients with BMETS from NSCLC provided no substantial value in quality of life, survival or reduction in steroid use when compared to dexamethasone and best supportive care [17]. The secondary analysis of a randomized clinical trial comparing post-operative WBRT and SRS revealed that WBRT was associated with more meaningful cognitive impairment and worse quality-of-life than SRS [18]. Hippocampal avoidance-WBRT (HA-WBRT) results in less cognitive impairment than WBRT, even in long-term survivors, without affecting the overall survival [16,19]. Thorough multidisciplinary consideration is required before recommending WBRT for patients with BMETS; if patients are in U-RPA classes 2B and 3 there may be no benefit in survival, and if in classes 1 and 2A, HA-WBRT would be desirable. The U-RPA may inform patients, physicians and multi-disciplinary brain metastasis boards in routine modern clinical practice of when WBRT may provide value to care [20,21].

In the 148 patients in U-RPA class 3 who received BDT, early palliative care consultation may have been valuable for patients as they make therapeutic decisions. Current treatment guidelines from ASCO-SNO-ASTRO emphasize early palliative care involvement in the management of patients with BMETS and poor prognosis [22,23,24]. Early (within eight weeks from diagnosis) rather than late palliative care consultation in patients with BMETS results in fewer inpatient admissions and emergency room visits as well as fewer diagnostic imaging studies and radiosurgery procedures [25]. Palliative care specialists involved as part of the multidisciplinary group at the time of diagnosis may help patients in U-RPA class 3 clarify the expectations about BDT and life expectancy, while reducing health care utilization.

We included only patients treated at academic institutions and found no significant difference between the facilities in survival for patients who received BDT. A retrospective analysis of the National Cancer Database revealed that patients treated at academic centers had significantly better survival than those treated at non-academic facilities [26]. Although we showed that the training and internal validation set had similar ROC curves, the U-RPA will require external validation using a cohort that includes non-academic facilities and sites outside the US. We emphasize that there should be an ongoing effort by health systems caring for patients with BMETS to collect prospective data on outcomes according to prognostic indices, such as the U-RPA, to improve the value of care provided to this growing population.

Our study has limitations inherent to retrospective abstraction of the data from the EMR. The KPS determined at the time of diagnosis does not consider improvement after steroids or surgery that could have changed the corresponding U-RPA class. We were unable to establish if the cause of death was secondary to the BMETS or systemic cancer. Survival was the only outcome measurement, but, as patients with BMETS live longer, integration of other outcomes such as CNS-progression-free survival and patient reported outcomes in prognostic indices would be of value when deciding about treatment. We did not compare the U-RPA with other prognostic indices because the goal of our study was to propose a tool that is easily applicable in the clinic to guide treatment decisions and reflects outcomes from contemporary care. Other prognostic scores such as the ds-GPA are more appropriate to use in the design and eligibility determination of clinical trials including patients with BMETS [8].

## 5. Conclusions

From the patients’, clinicians’, and health systems’ perspectives, BMETS value-based care requires a standardized, validated and practical prognostic index that applies to outcomes from contemporary treatment paradigms. Based on our study, the U-RPA may fulfill the need in modern times, but to continue to be relevant, the tool would demand future adjustments as more effective systemic cancer treatments are available. The U-RPA can inform patients and clinicians at the time of BDT decisions, specialty societies in providing evidence-based guideline recommendations and health systems by offering personalized benchmarks for quality improvement strategies. Furthermore, the growing number of integrated multidisciplinary teams specialized in the attention of patients with brain metastases reflects the complexities of care and the availability of more effective systemic treatment options [20,21,27,28]. Such programs will require uniform and easy to measure prognostic tools to make treatment decisions that adhere to accepted guideline recommendations. If we are to provide value care to patients with BMETS, it is of the utmost relevance to consistently estimate and document in the medical record a prognostic indicator that can identify those who will benefit from BDT. Our study supports determining and documenting the U-RPA at the time of BMETS diagnosis as part of contemporary routine practice, allowing the clinician to communicate to the patient and caregivers an estimated survival outcome that will aid in informed, value-driven therapeutic shared decision-making.

## Figures and Tables

**Table 1:** Demographic characteristics and treatment for all patients.

Variable		Total Number (862 Patients)
Median age years (range)		65 (23–93)
Median KPS (range)		80 (20–100)
Sex		
	Female (%)	420 (49%)
	Male (%)	442 (51%)
Primary Tumor type		
	NSCLC (%)	393 (46%)
	Melanoma (%)	96 (11%)
	Breast (%)	94 (11%)
	SCLC (%)	63 (7%)
	Renal (%)	53 (6%)
	Others (%)	163 (19%)
RPA		
	I (%)	84 (10%)
	II (%)	570 (66%)
	III (%)	208 (24%)
U-RPA		
	1 (%)	84 (10%)
	2A (%)	259(30%)
	2B (%)	311 (36%)
	3 (%)	208 (24%)
Treatment		
	SRS (70 after surgery)	371 (43%)
	WBRT (41 after surgery)	305 (35%)
	SMT (9 after surgery)	76 (9%)
	BSC (20 had surgery)	110 (13%)

KPS: Karnofsky Performance Score; NSCLC: non-small cell lung cancer, SCLC: small cell lung cancer; RPA Recursive Partition Analysis; U-RPA: Updated-RPA; SRS: stereotactic radiosurgery; WBRT: whole brain radiation therapy; SMT: systemic medical therapy; BSC: best supportive care.

**Table 2:** Survival estimates in months according to RPA, U-RPA and years patients were treated.

Class	RPA *	RPA	U-RPA
	1979–1993	2017–2019	2017–2019
	*n* = 1200	*n* = 752	*n* = 752
1	7.4	28.1	28.1
2A	4.2 *	10.9 *	14.7
2B			7.6
3	2.3	3.3	3.3

RPA: Recursive Partitioning Analysis, U-RPA: Updated Recursive Partitioning Analysis, * RPA class 2, Reference [9].

**Table 3:** Hazard ratios (HR) for multivariate analysis showing 95% confidence intervals (CI) and significance in relation to survival.

	HR	95.0% CI		*p*-Value
		Lower	Upper	
U-RPA 1				0.001
U-RPA 2A	1.63	1.16	2.31	0.005
U-RPA 2B	1.81	1.27	2.58	<0.001
U-RPA 3	2.5	1.5	4.16	<0.001
Age	1.01	1	1.02	0.006
SRS	0.85	0.7	1.02	0.078
WBRT	0.81	0.72	0.92	0.002
KPS	0.99	0.97	1	0.013
Surgery	1.11	0.99	1.24	0.073

**Table 4:** Median survival of patients according to primary cancer and U-RPA.

Primary	No.	U-RPA CLASS				Log-Rank
		1	2A	2B	3	*p* Value
NSCLC	345	43.1	15.8	8.1	3.5	<0.0001
NSCLC *	283	43.1	14.0	7.7	3.5	<0.0001
Melanoma	86	47.6	19.3	4.3	2.3	<0.0001
Breast	83	46.8	28.5	8.4	3.2	<0.0001

NSLC: Non-small cell lung cancer, U-RPA:, * NSCLC excluding patients with targetable mutation.

## Data Availability

The data from this study may be made available upon request sent to the corresponding author who will review and seek approval from study investigators.

## Supplementary Materials

The following supporting information can be downloaded at: https://www.mdpi.com/article/10.3390/cancers15123255/s1, Figure S1: Kaplan-Meier survival curves by primary cancer (A) NSCLC, (B) NSCLC without genomic alterations, (C) melanoma, and (D) breast cancer; Figure S2: Kaplan Meier survival curves by academic center (A) all patients receiving BDT (B) patients receiving WBRT and (C) patients receiving SRS. Table S1: Median survival of patients according to treatment and U-RPA.
