# Supplementary material for: Relevance of the Updated Recursive Partitioning Analysis (U-RPA) Classification in the Contemporary Care of Patients with Brain Metastases
Source: Cancers (Basel). 2023 Jun 20;15(12):3255. doi: 10.3390/cancers15123255 (PMC10297002; doi:10.3390/cancers15123255)
Supplement: Supplementary file 1 [file cancers-15-03255-s001.zip › cancers-2412512-supplementary.pdf]

Table S1. Median survival of patients according to treatment and U-RPA.

Class	U-RPA BDT N=752	SRS N=371	WBRT N=305	SMT N=76	Surgery* N=120
1	28.1	29.1	22.4	Undefined	25.4
2A	14.7	16	9.6	30.9	21.6
2B	7.6	10.9	4.9	6.2	8.4
3	3.3	4	2.9	3.1	5.7

U-RPA: Updated-RPA; BDT: brain-directed therapy SRS: stereotactic radiosurgery; WBRT: whole-brain radiation therapy; SMT: systemic medical therapy* Surgery followed by SRS, WBRT or SMT.

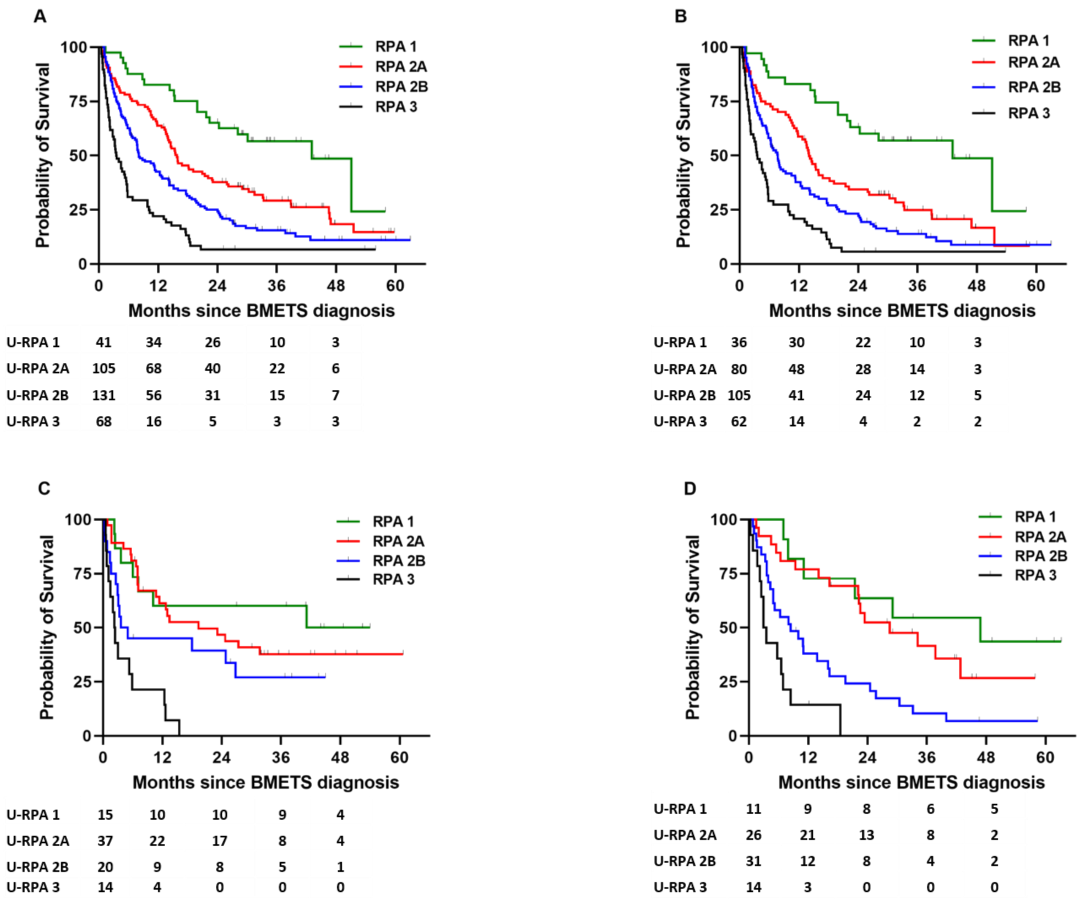

Figure S1. Kaplan-Meier survival curves by primary cancer A) NSCLC, B) NSCLC without genomic alterations, C) melanoma, and D) breast cancer.

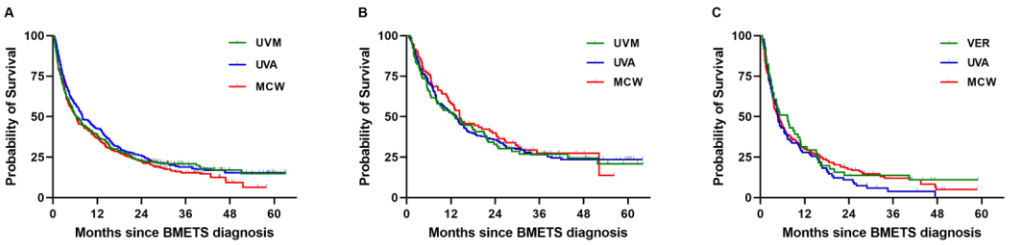

UVM	220	85	50	23	10
UVA	331	142	86	49	18
MCW	310	109	62	27	6

UVM	79	41	26	13	10
UVA	196	104	71	43	19
MCW	96	52	35	14	3

UVM	51	17	8	6	2
UVA	86	25	10	3	0
MCW	168	50	26	14	4

Figure S2. Kaplan Meier survival curves by academic center A) all patients receiving BDT B) patients receiving WBRT and C) patients receiving SRS.
